# A Swedish Population‐Based Study Found That Muscle Hypertonia in Children With Cerebral Palsy Varied by Age, Subtype, Functional Level, and Muscle Group

**DOI:** 10.1111/apa.70649

**Published:** 2026-06-13

**Authors:** Gunnar. Hägglund, Ann. I. Alriksson‐Schmidt

**Affiliations:** ^1^ Department of Clinical Sciences Lund University Lund Sweden; ^2^ Department of Orthopaedics Skåne University Hospital Lund Sweden; ^3^ Lund Disability Research Hub Lund Sweden

**Keywords:** Ashworth scale, cerebral palsy, muscle tone, registry study, spasticity

## Abstract

**Aim:**

We examined how lower limb muscle tone was related to age, sex, Gross Motor Function Classification System (GMFCS) level, and the cerebral palsy (CP) subtype in Swedish children with CP.

**Methods:**

This registry‐based cohort study from 2023 to 2024 used data on all 3 216 children aged 1–15 from the Swedish follow‐up program and national CP Registry. Muscle tone was assessed with the Ashworth Scale in the gastrocsoleus, hamstring, and adductor muscles. Associations between muscle tone and age, GMFCS level, sex, and CP subtype were analysed using descriptive statistics and logistic regression.

**Results:**

Hypertonia, defined as Ashworth grade ≥ 1, was present in the gastrocsoleus in 84% of the children, in the hamstrings in 52%, and in the adductors in 34%. Prevalence increased with higher GMFCS levels and varied across age and muscle groups. Gastrocsoleus hypertonia peaked at 7–9 years and then declined. Hamstring hypertonia increased steadily up to 13–15 years. Adductor hypertonia showed a U‐shaped pattern across age groups. Children with bilateral spastic or dyskinetic CP had the highest hypertonia levels in all muscle groups.

**Conclusion:**

Muscle hypertonia in children with CP varied by age, GMFCS level, CP subtype, and muscle group. This should be considered when planning treatment strategies.

## Key Clinical Message

- Understanding how muscle hypertonia varies by age, function, subtype, and muscle group is important when it comes to providing individualised management for cerebral palsy.
- This population‐based study of 3 216 children found that hypertonia varied by muscle group, increased with higher Gross Motor Function Classification System levels, and showed age‐related differences across subtypes.
- Specific treatment should focus on age, muscle, and function strategies and include long‐term clinical monitoring.

**Abbreviations**
AS: Ashworth Scale
CI: confidence interval
CP: cerebral palsy
CPUP: Swedish combined follow‐up program and national registry for individuals with CP
GMFCS: Gross Motor Function Classification System
OR: odds ratio

## 1 Introduction

Spasticity is a dominant symptom in many individuals with cerebral palsy (CP). It is defined as a velocity‐dependent increase in muscle tone, namely hypertonia, resulting from hyperexcitability of the stretch reflex [1]. In some children with CP, spasticity may partially compensate for underlying muscle weakness. However, it has also been shown to impair mobility, function, and participation in daily activities [2]. Studies have also demonstrated that elevated muscle tone can negatively impact musculoskeletal development, leading to muscle contractures, torsional deformities, hip dislocation, and scoliosis [3, 4]. In addition, spasticity has been commonly associated with pain [5].

Spasticity is common in the spastic subtypes of CP, but it may also occur in the dyskinetic, ataxic, and mixed subtypes [6]. Its prevalence and severity have varied across muscle groups and with age [7, 8]. There are various treatment approaches to reducing spasticity, including both local and systemic treatments, which may be either reversible or permanent. Treatment with botulinum toxin A provides transient tone reduction in the muscles that are being injected. Selective dorsal rhizotomy permanently reduces muscle tone by selectively cutting sensory nerve rootlets that contribute to spasticity. Baclofen, administered either orally or via an intrathecal pump, provides reversible tone reduction. In addition, several other medications can be used to achieve overall and reversible reductions in muscle tone. To select appropriate treatment methods, it is important to understand how muscle tone is distributed among different muscle groups and age ranges in children with various CP subtypes and functional levels.

The Swedish combined follow‐up program and national CP Registry for individuals with CP (CPUP) was initiated in southern Sweden in 1994. Its overall purpose was to enable early identification and treatment of secondary complications of CP through standardised follow‐up. CPUP currently includes more than 95% of individuals with CP born in Sweden in 2000 or later [9]. The program includes regular, standardised assessments, including evaluation of muscle tone using the Modified Ashworth Scale (AS) [10].

The aim of this study was to examine the degree of muscle tone in different lower limb muscle groups in all children with CP in Sweden. We wanted to do this in relation to age, Gross Motor Function Classification System (GMFCS) [11] level, and CP subtype.

## 2 Patients and Methods

This population‐based registry study used data on 3 216 children up to 15 years old who were reported to the CPUP between January 1, 2023, and December 31, 2024. Diagnoses of CP were typically confirmed by child neurologists in accordance with the Surveillance of Cerebral Palsy in Europe network [6]. They were classified as bilateral, unilateral spastic, dyskinetic, ataxic, and mixed or non‐classifiable. Classification was based on the dominant symptom, which meant that children who did not have spastic CP still exhibited varying degrees of spasticity.

The children had undergone regular, standardised examinations by their local physical therapists, following the protocols established by the CPUP [9, 12]. The physical therapists classified gross motor function according to GMFCS levels. Children at GMFCS level I were assessed annually until six years of age, and every second year after that. Children with GMFCS levels II–V were assessed twice a year until six years of age, and annually after that. The examination included muscle tone measurements, according to the modified AS (Table 1) [10]. This assessment is standardised and carried out in accordance with the CPUP manual [12].

**TABLE 1:** The modified Ashworth scale [10].

Score	Description of the muscle tone
0	Normal tone, no increase in tone
1	Slight increase in tone: A catch and release at the end of the range of motion
1+	Slight increase in tone: Catch, followed by minimal resistance in the remainder of the range
2	More marked increase in tone through most of the range
3	Considerable increase in tone, passive movement difficult
4	Affected parts rigid in flexion or extension

All children enrolled in the CPUP who were 15 years of age or younger in 2023 or 2024 were eligible for inclusion. Those who had undergone selective dorsal rhizotomy, or had an intrathecal baclofen pump, were excluded. Children treated with botulinum toxin A were included. Data on age, sex, subtype, and GMFCS level were extracted from the CPUP database. The subject's age at the time of the examination was calculated, rounded to whole years, grouped into intervals of three years and treated as a categorical variable. If children had more than one assessment during the time period we studied, we used their age, sex, and the first GMFCS level reported. Recorded levels of muscle tone in the gastrocsoleus, hamstrings, and adductor muscles were extracted. Data from the side with the highest degree of muscle tone were used for each child. Levels 1 and 1+ were combined into one level, corresponding to the original AS [13].

### 2.1 Data Analysis

Distributions of the data were inspected, and continuous variables are presented as means and standard deviations, and categorical variables as numbers and percentages. The degree of muscle tone related to age, sex, CP subtype, and GMFCS level was analysed using logistic regression with odds ratios (OR). The analyses were performed using R version 4.5.2 (R Foundation, Vienna, Austria).

## 3 Results

Data on 3 306 children aged 1–15 years were reported to the CPUP register in 2023–2024 and initially included in the study. We excluded 90 children, as 57 had undergone selective dorsal rhizotomy and 33 had intrathecal baclofen pumps and were not eligible for inclusion. The remaining 3 216 (59% boys) children were included in the analyses. The distribution of age, sex, GMFCS levels, and CP subtypes is presented in Table 2. The CP subtypes were missing for 26% of the children, and 426 (13%) of these were four years of age or younger.

**TABLE 2:** Distribution of age, sex, GMFCS level and CP subtype.

	Boys *n* (%)	Girls *n* (%)	Sex not reported (*n*)	Total *n* (%)
Age (years)				
1–3	328 (17)	235 (17)	0	563 (18)
4–6	367 (20)	246 (19)	3	616 (19)
7–9	383 (20)	239 (18)	3	625 (19)
10–12	430 (23)	322 (24)	5	757 (24)
13–15	372 (20)	282 (22)	1	655 (20)
GMFCS level				
I	897 (47)	604 (46)	5	1 506 (47)
II	342 (18)	235 (18)	2	579 (18)
III	139 (7)	112 (8)	1	252 (8)
IV	242 (13)	184 (14)	1	427 (13)
V	260 (14)	189 (14)	3	452 (14)
CP‐subtype				
Unilateral spastic	556 (30)	417 (31)	3	976 (30)
Bilateral spastic	579 (31)	398 (30)	1	978 (30)
Dyskinetic	166 (9)	110 (8)	2	278 (9)
Ataxic	32 (2)	40 (3)	0	72 (2)
Mixed	43 (2)	36 (3)	0	79 (2)
Unclassified	504 (27)	323 (24)	6	833 (26)*
Total	1 880 (59)	1 324 (41)	12	3 216 (100)

Abbreviations: CP = Cerebral palsy; GMFCS = Gross Motor Function Classification System.

* 426 children were four years of age or younger.

When all 3 216 children were analysed, 82% had muscle hypertonia, defined as any AS level of 1–4 in the gastrocsoleus muscles: 50% in the hamstring muscles, and 34% in the adductor muscles (Table 3). AS grade 4 was reported in 23 children in the gastrocsoleus muscles, three children in the hamstring muscles, and eight in the adductor muscles. Due to the small numbers, grades 3 and 4 were combined in the subsequent analyses.

**TABLE 3:** Degree of muscle hypertonia related to sex and muscle groups.

Ashworth Scale (AS)	Boys *n* (%)	Girls *n* (%)	Sex not reported (*n*)	Total *n* (%)
Gastrocsoleus				
0	306 (16)	226 (17)	2	534 (17)
1	977 (52)	662 (50)	5	1 644 (51)
2	281 (15)	193 (15)	2	476 (15)
3	107 (6)	84 (6)	2	193 (6)
4	11 (1)	12 (1)	0	23 (1)
AS not reported	198 (11)	147 (11)	1	346 (11)
Hamstrings				
0	818 (44)	574 (43)	4	1 396 (43)
1	618 (33)	448 (34)	5	1 071 (33)
2	147 (8)	93 (7)	2	242 (8)
3	45 (2)	33 (2)	0	78 (2)
4	3 (0)	0 (0)	0	3 (0)
AS not reported	249 (13)	176 (13)	1	426 (13)
Adductors				
0	1 064 (57)	771 (58)	5	1 840 (57)
1	396 (21)	255 (19)	4	655 (20)
2	100 (5)	73 (6)	2	175 (5)
3	35 (2)	26 (2)	0	61 (2)
4	6 (0)	2 (0)	0	8 (0)
AS not reported	279 (15)	197 (15)	1	477 (15)
Total	1 880	1 324	12	3 216 (100)

Abbreviation: GMFCS, Gross Motor Function Classification System.

**FIGURE 1:**
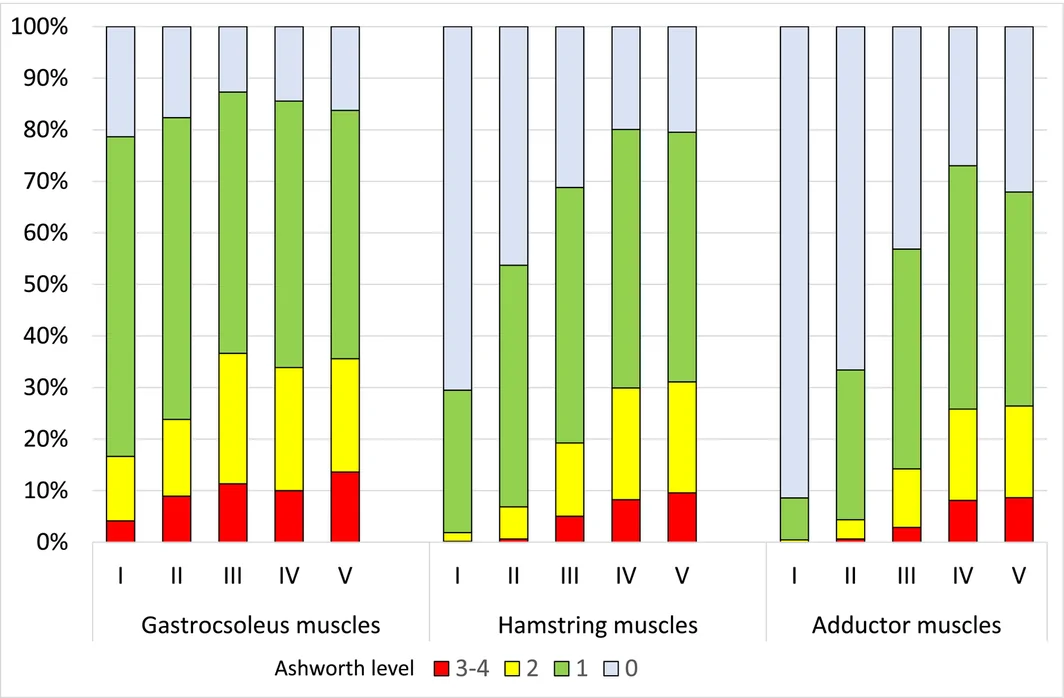
Degree of muscle tone in the gastrocsoleus, hamstrings, and adductors according to the Ashworth Scale, stratified by the Gross Motor Function Classification System level. The Y‐axis is the proportion of children with reported degrees of hypertonia, according to the Ashworth Scale.

There was no statistically significant difference in muscle tone levels between the sexes (Table 3). The prevalence and severity of hypertonia increased with higher GMFCS levels across all muscle groups (Figure 1). However, only a small difference was observed between GMFCS levels IV and V. The proportion with AS ≥ 2 in the gastrocsoleus muscle increased from 17% in those with GMFCS I to 37% with GMFCS IV. Corresponding increases were observed for the hamstrings (2% to 30%) and for the adductors (1% to 27%).

**FIGURE 2:**
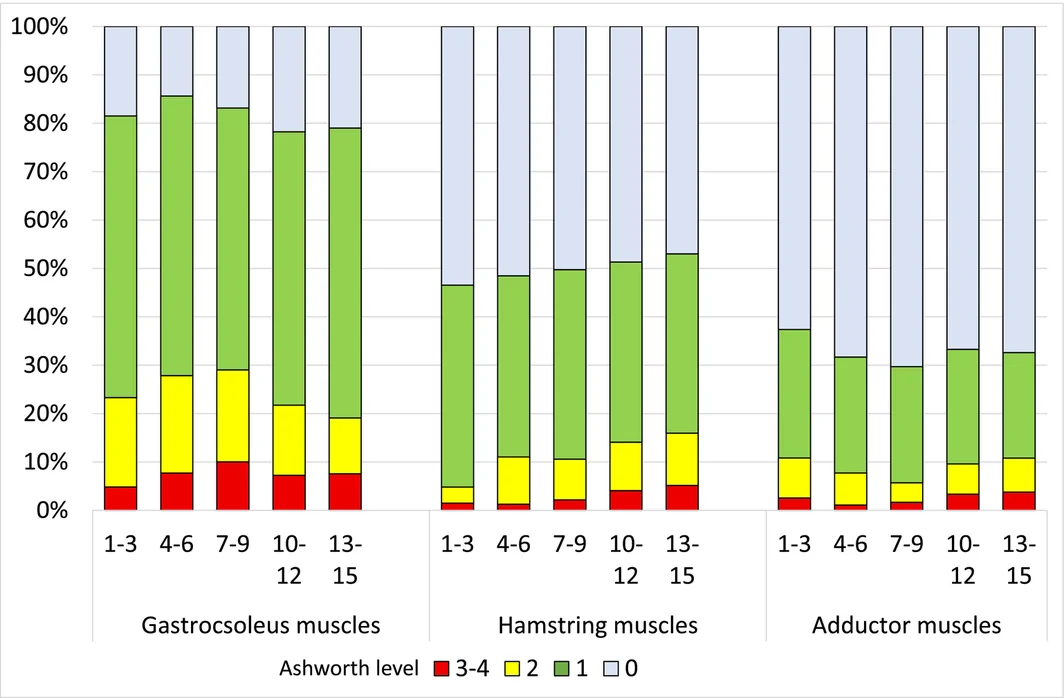
Degree of muscle tone in the gastrocsoleus, hamstrings, and adductors, according to the Ashworth Scale and related to age. The Y‐axis is the proportion of children with reported degrees of hypertonia, according to the Ashworth Scale.

Hypertonia trends varied by muscle group (Figure 2). The proportion of children with AS ≥ 2 in the gastrocsoleus muscle increased from 23% at 1–3 years of age to 28% at 7–9 years and then decreased to 18% at 13–15 years. Hamstring hypertonia increased with age, from 5% at 1–3 years of age to 17% at 13–15 years. Adductor hypertonia decreased from 11% at 1–3 years of age to 6% at 7–9 years, then increased to 11% at 13–15 years.

**FIGURE 3:**
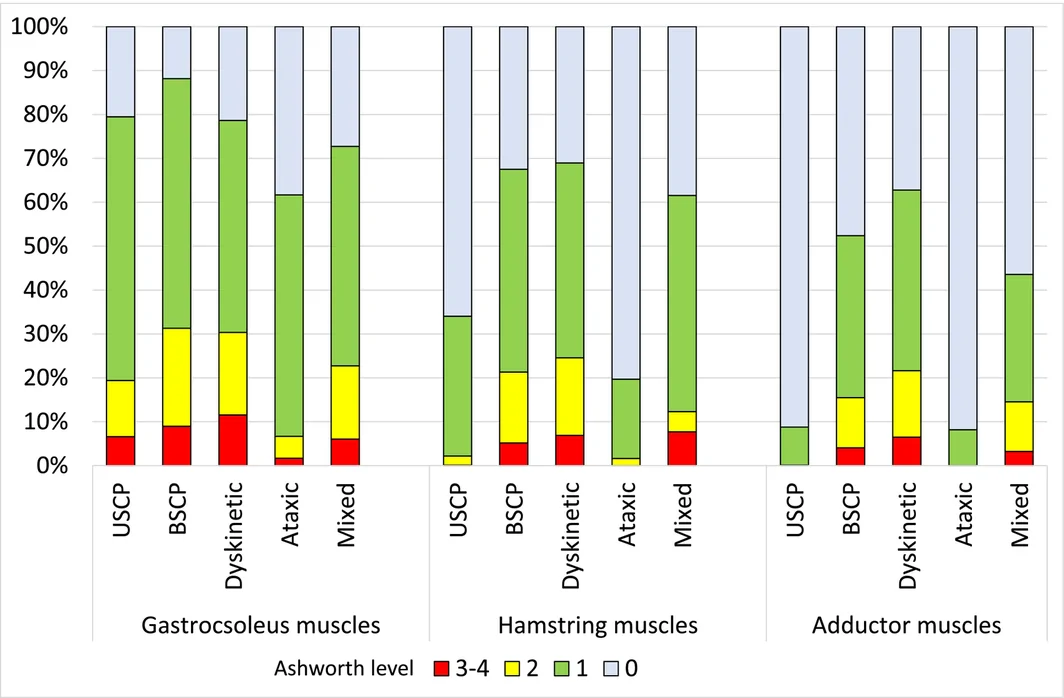
Degree of muscle tone in the gastrocsoleus, hamstrings, and adductors, according to the Ashworth scale and related to the CP subtype. The Y‐axis is the proportion of children with reported degrees of hypertonia according to the Ashworth scale. USCP, unilateral spastic cerebral palsy; BSCP, bilateral spastic cerebral palsy.

Children with bilateral spastic CP and dyskinetic CP exhibited the highest hypertonia across all muscle groups (Figure 3). Children with unilateral spastic CP showed lower gastrocsoleus tone, namely 22% with AS ≥ 2, compared to 38%–40% with bilateral spastic CP or dyskinetic CP. There was almost no AS ≥ 2 in hamstrings or adductors. Children with ataxic CP demonstrated minimally increased tone, while those with mixed CP displayed intermediate levels.

The logistic regression analysis showed that age and GMFCS level were independently statistically significant factors that influenced the proportion of children with hypertonia (AS > 2) in all three muscle groups. In addition, the CP subtype showed a statistically significant association with hypertonia in the hamstring muscles, but not in the gastrocsoleus or adductor muscles (Table 4).

**TABLE 4:** Hypertonia AS 2 or higher, related to age, GMFCS level, and CP subtype in gastrocsoleus, hamstrings, and adductor muscles.

Variable (reference group)	Gastrocsoleus	Hamstrings	Adductors
	OR (95% CIs)/*p*	OR (95% CIs)/*p*	OR (95% CIs) *p*
Age (1–3 years of age)	< 0.001	< 0.001	< 0.05
2004–6 years of age	1.07 (0.69–1.68)	4.38 (1.79–13.19)	1.39 (0.66–3.10)
7–9 years of age	1.04 (0.68–1.63)	3.91 (1.61–11.71)	0.68 (0.32–1.55)
10–12 years of age	0.71 (0.46–1.10)	5.64 (2.37; 16.67)	1.40 (0.69–3.05)
13–15 years of age	0.59 (0.38–0.93)	7.08 (2.97–21.00)	1.54 (0.75–3.37)
GMFCS level (Level I)	< 0.001	< 0.001	< 0.001
Level II	1.43 (1.04–1.95)	2.66 (1.45–4.95)	5.52 (1.98–19.55)
Level III	2.54 (1.67–3.86)	8.27 (4.42; 15.81)	12.9 (4.73–45.14)
Level IV	2.30 (1.60–3.31)	13.5 (7.69–24.51)	26.3 (10.5–88.25)
Level V	2.33 (1.61–3.38)	12.3 (6.98–22.53)	23.0 (9.18–77.12)
CP‐subtype^a^ Unilateral spastic	> 0.05	< 0.001	> 0.05
Bilateral spastic cerebral palsy	1.23 (0.93–1.62)	3.09 (1.77–5.58)	^b^
Dyskinetic CP	0.96 (0.63–1.45)	2.06 (1.08–4.05)	0.84 (0.56–1.23)

Abbreviations: AS = Ashworth Scale, CIs = confidence intervals, GMFCS = Gross Motor Function Classification System, OR = Odds ratio, Unilateral spastic CP = Unilateral spastic cerebral palsy, bilateral spastic CP = Bilateral spastic cerebral palsy.

^a^ Only children with unilateral spastic, bilateral spastic, and dyskinetic CP were included in the analysis, due to the small number of participants with ataxic or mixed subtypes.

^b^ Only one child had unilateral spastic CP and AS 2 or higher.

## 4 Discussion

This study demonstrated that muscle hypertonia was highly prevalent and affected most children with CP. Its distribution and severity varied significantly by age, GMFCS level, and CP subtype. As expected, higher GMFCS levels were associated with increased hypertonia. This was most prevalent in the gastrocsoleus muscle, followed by the hamstrings and adductor muscles. Children with bilateral spastic CP and dyskinetic CP showed the highest levels of hypertonia across all muscle groups.

The high degree of hypertonia in the hamstring and adductor muscles in GMFCS levels IV and V corresponded with the predominance of children with bilateral spastic CP and dyskinetic CP at these GMFCS levels. Children with unilateral spastic CP, who are often classified at GMFCS levels I–II, corresponded to the prevalence of hypertonia, with dominant increased tone in the gastrocsoleus muscle.

The progressive increase in hypertonia that children with CP experience during the early years of their life has been well documented in numerous studies [7, 14, 15, 16, 17]. Cross‐sectional studies from Sweden have demonstrated a shift in the age at which the highest proportion of children exhibited hypertonia in the gastrosoleus muscles. This peak occurred at approximately four years of age in children born from 1995 to 2006 [16], and at 5.5 years in those born between 2000 to 2015 [7]. It was 7–9 years of age in our study. The proportion of children with AS 2 or higher at the peak age decreased from 47% to 38% to 28% in the same studies. This trend needs to be analysed in future longitudinal studies.

The classification of CP subtypes, according to the Surveillance of Cerebral Palsy Network in Europe, is based on the most dominant symptom. Many children with dyskinetic CP also have spasticity [18]. The ataxic subtype includes children with ataxic diplegia, which explains why some children in this subgroup had increased muscle tone. Children with bilateral spastic CP and dyskinetic CP had significantly higher levels of hypertonia than other subtypes, reflecting the broader motor involvement in these groups. Children with unilateral spastic CP showed relatively low levels of hypertonia, particularly in proximal muscle groups.

The results were consistent with a previous study that analysed botulinum toxin A treatment in the same cohort [19]. This was most frequently administered to the gastrocsoleus muscles, particularly in children aged 7–9 years and in those with bilateral spastic cerebral palsy.

Children treated with botulinum toxin A were included in our analyses. At the time of our data analysis, 26% had already received botulinum toxin, and in many cases, this treatment had been provided more than three months earlier. It cannot be ruled out that some children may have presented with lower muscle tone than they would have done if they had not received treatment. However, the treatment patterns for botulinum toxin A were consistent with the hypertonia profile for age, CP subtype, and GMFCS level. This means that any treatment effect would probably have attenuated, rather than exaggerated, the differences observed between the groups. Any treatment effect would only have reduced the observed differences among the groups.

The AS is the most widely used instrument for assessing muscle tone. Some studies have shown that the scale has limited interrater reliability [20]. However, a systematic review and meta‐analysis concluded that inter‐rater and intra‐rater agreements were satisfactory [21]. Reliability was slightly higher for the original AS, which was used in the present study [20]. Any issues were further reduced by grouping AS levels 2–4 together in the analysis. In our study, low reliability would have just increased the observed variation in the population AS measurements in the absence of systematic errors. This would only have affected our ability to correctly detect an actual change.

The validity of the AS has been analysed by several studies [22]. The modified AS quantifies resistance to passive movements, including both viscoelastic properties of soft tissue and neuronal activity. It is therefore recommended that the instrument be described as a measurement of muscle tone, rather than a specific measurement of spasticity. Despite this, the modified AS is the most common instrument for analysing spasticity, e.g., after spasticity‐reducing treatments. Some studies that have compared spasticity measurement with the modified AS with laboratory techniques, including electromyography recordings, have shown good correlations between the instruments [23, 24].

The findings in this study have several clinical implications. The large variations in the presence and degree of hypertonia related to muscle groups, age, CP subtype, and GMFCS levels can support clinical decision‐making when planning tone‐reducing treatment. In particular, these insights may guide the choice between local and systemic interventions, as well as between temporary and permanent treatment options.

### 4.1 Strengths and Limitations

The study had notable strengths, including the large sample size and the population‐based design, which provided almost complete coverage of the population that had undergone standardised assessments. These enhanced the generalisability and external validity of the findings.

There were several limitations to this study. The CP subtype classification was incomplete for a substantial number of children. In addition, the cross‐sectional design made it impossible to analyse the longitudinal development of hypertonia with age.

## 5 Conclusion

This Swedish study showed that the distribution and degree of muscle tone in children with CP varied significantly by age, GMFCS level, CP subtype, and the muscle groups that were affected. It is important to consider this information when selecting treatment methods to reduce muscle tone in children with CP.

## Author Contributions

**Ann. I. Alriksson‐Schmidt:** conceptualization, investigation, methodology, writing – review and editing, formal analysis. **Gunnar. Hägglund:** conceptualization, investigation, writing – original draft, formal analysis, methodology, writing – review and editing.

## Funding

This study received financial support from Stiftelsen för Bistånd åt Rörelsehindrade i Skåne, the Swedish Research Council for Health, Working Life, and Welfare (2018‐01468), and Alfred Österlunds Stiftelse. None of the funders were involved in the study or paper.

## Ethics Statement

This study was conducted in accordance with the Declaration of Helsinki and approved by the Swedish Ethical Review Authority (Reg. no. 2025–00145‐01). Permission was obtained to extract data from the CPUP registry.

## Consent

Informed consent to use data for research was provided by all families participating in the CPUP.

## Conflicts of Interest

The authors declare no conflicts of interest.

## Data Availability

The data that support the findings of this study are available on request from the corresponding author. The data are not publicly available due to privacy or ethical restrictions.
